# Alleviation of High-Fat Diet-Induced Hyperlipidemia in Mice by *Stachys sieboldii* Miq. Huangjiu via the Modulation of Gut Microbiota Composition and Metabolic Function

**DOI:** 10.3390/foods13152360

**Published:** 2024-07-26

**Authors:** Jingzhang Geng, Yunxia Wu, Honglei Tian, Jianwei Dong

**Affiliations:** 1 Shaanxi Province Key Laboratory of Bio-Resources, QinLing-Bashan Mountains Bioresources Comprehensive Development C. I. C., Qinba State Key Laboratory of Biological Resources and Ecological Environment, Shaanxi University of Technology, 1 East 1st Ring Road, Hanzhong 723001, China; gengjingzhang@163.com (J.G.); zshanshan2024@163.com (Y.W.); thl0993@sina.com (H.T.); 2 School of Life Science and Technology, Xi’an Jiaotong University, Xianning West Road, Xi’an 710049, China

**Keywords:** *Stachys sieboldii* Miq. Huangjiu, hyperlipidemia, gut microbiota, metabolic

## Abstract

Hyperlipidemia is a chronic disease that is difficult to cure, and long-term pharmacotherapy may have negative consequences. Dietary therapy is a very promising strategy, and Chinese rice wine (Huangjiu) will play an important role because of its many biologically active components. In this work, the alleviating effect of *Stachys sieboldii* Miq. Huangjiu (CSCHJ) on high-fat diet-induced hyperlipidemia in mice was investigated, which is brewed from the wheat Qu with the addition of *Stachys sieboldii* Miq. and contains 15.54 g/L of polysaccharides. The experimental results showed that CSCHJ inhibited appetite, reduced body weight and blood sugar levels, and downregulated the serum levels of total cholesterol (TC), triglycerides (TG), and low-density lipoprotein cholesterol (LDL-C) while concurrently upregulating high-density lipoprotein cholesterol (HDL-C) in the high-fat diet-induced hyperlipidemia mice. At the same time, it was discovered that alcohol worsens hyperlipidemia symptoms and related physiological markers, implying that CSCHJ polysaccharides may play a role in hyperlipidemia treatment. Through the assessment of organ indices, liver and kidney function, and tissue staining, CSCHJ demonstrated efficacy in repairing liver, kidney, and colon mucosal damage in hyperlipidemic mice. Furthermore, 16S rDNA sequencing and gas chromatography studies revealed that CSCHJ effectively restored the intestinal microbial structure and enhanced the quantity of fecal short-chain fatty acids (SCFAs) in hyperlipidemic mice. Therefore, the alleviating effect of CSCHJ on hyperlipidemia in mice may be attributed to its regulation of energy metabolism by repairing liver, kidney, and colon mucosal damage and restoring the gut microbiota structure, among other mechanisms. Overall, our findings provide evidence that CSCHJ contains active ingredients capable of alleviating hyperlipidemia, thereby laying a theoretical foundation for the extraction of bioactive substances from Huangjiu for future medical or dietary use.

## 1. Introduction

Hyperlipidemia (HLP) arises from aberrant lipid metabolism, which is characterized by decreased levels of high-density lipoprotein cholesterol (HDL-C) and raised levels of triglycerides (TG), total cholesterol (TC), and low-density lipoprotein cholesterol (LDL-C). Presently, HLP serves as a precursor to various ailments, including liver and kidney injury, type 2 diabetes (T2D), hypertension, atherosclerosis, and other metabolic disorders [1,2,3], significantly impinging on human well-being. In clinical settings, the treatment of hyperlipidemia predominantly relies on statins and fibrates. Despite their notable therapeutic efficacy, prolonged administration is requisite. This protracted drug regimen, coupled with substantial dosages, engenders a spectrum of adverse effects, ranging from nausea and vomiting to potentially inducing hepatic impairment [4,5,6]. Consequently, the quest for safe and efficacious hypolipidemic agents has emerged as a paramount research endeavor.

Chinese rice wine (Huangjiu), an integral component of traditional fermented fare, boasts a brewing legacy spanning over 5000 years [7]. It stands as a quintessential example of low-alcohol grain fermentation, crafted predominantly from cereals as raw materials, wheat Qu as a saccharifying agent, and yeast as a starter culture [8,9]. Revered for its exquisite flavor, nuanced taste, and abundant nutritional profile, Huangjiu is distinguished by its richness in diverse bioactive compounds conducive to health. Notably, it harbors polysaccharides, oligosaccharides, free amino acids, peptides, organic acids, polyphenols, and trace minerals [10,11], each imparting beneficial functions encompassing gut microbiota enhancement, antioxidation, anti-inflammation, hypoglycemia mitigation, lipid reduction, blood pressure moderation, hepatoprotection, and immune augmentation [12,13,14].

The functional constituents present in Huangjiu intricately intertwine with the intricate microbial metabolism within the fermentation milieu [15,16]. Consequently, the microbial community structure of Jiuqu exerts a profound influence on the flavor profile and bioactive constituents of Huangjiu [17], underscoring the pivotal role of Jiuqu as the backbone of wine production. Leveraging prebiotics derived from the Chinese herbal medicine *Stachys sieboldii* Miq., our research group has endeavored to enhance the microbial community structure of Jiuqu. Through this intervention, we have successfully brewed *Stachys sieboldii* Miq. Huangjiu (CSCHJ) enriched with polysaccharides—an essential bioactive component of Huangjiu [18]. We hypothesize that CSCHJ, owing to its elevated polysaccharide content, will exhibit noteworthy biological functionality. However, to date, no pertinent research endeavors have been undertaken to explore the biological activities of CSCHJ.

This study aims to elucidate the lipid-lowering properties of CSCHJ using a high-fat diet-induced hyperlipidemia mouse model. Through the assessment of fundamental physiological parameters in experimental animals, including body weight, the levels of four blood lipids, the indicators of liver and kidney function, the content of intestinal SCFAs, and the diversity of gut microbiota, we seek to preliminarily investigate the mechanistic impact of CSCHJ on hyperlipidemia. The findings of this study are anticipated to furnish a theoretical framework and serve as a reference for the development of functional Huangjiu.

## 2. Materials and Methods

### 2.1. Materials and Reagents

*Stachys sieboldii* Miq. Huangjiu was prepared in our own laboratory. Edible alcohol was purchased from a local supermarket. Detection kits for TC, TG, LDL-C, HDL-C, glutamic oxaloacetic transaminase (AST), glutamic-pyruvic transaminase (ALT), serum creatinine (Scr), and blood urea nitrogen (BUN) were purchased from Nanjing Jiancheng Biotechnology Co., Ltd., Nanjing, China. Hematoxylin, eosin and polyformaldehyde were purchased from Wuhan Servicebio Technology Co., Ltd., Wuhan, China. The basic and high-fat diet used in the animal experiments were purchased from Medicience Ltd., Yangzhou, China. Potassium dichromate, phenol, potassium sodium tartrate, and sodium sulfite were purchased from Tianjin Damao Chemical Reagent Factory, Tianjin, China, and all chemical reagents are of analytical grade.

### 2.2. Animals and Experimental Design

Healthy male KM mice (5 weeks old, average weight 20 ± 2 g) were bought from Hunan SJA Laboratory Animal Co., Ltd. (Changsha, China) and housed under standard conditions (23 ± 2 °C, 60 ± 5% relative humidity, and a 12 h light/dark cycle) with free access to standard food and water. Following a week of acclimation, the mice were randomly assigned to groups for subsequent experiments. The food intake and body weight were measured weekly. These mice were randomly assigned to two groups: fifteen mice in the normal control group (NC), which received a typical diet, and sixty mice in the model group, which was fed a high-fat diet. The basic diet consisted mostly of chicken powder, fish powder, soybean flour, bran, corn, complex vitamins, and trace elements. The high-fat diet consisted of 1.5% cholesterol, 0.5% sodium gallate, 2% premix, 2% dicalcium phosphate, 12% casein, 10% lard, 3% sesame oil, 20% fructose, and 49% base plastic. Following a 12 h fast, blood samples were collected from both groups’ eye sockets by eyeball extripation after a 4-week feeding period. TC, TG, HDL-C, and LDL-C levels were all tested. The mouse hyperlipidemia model was successfully established, since there was a significant difference in the data between the two groups (*p* < 0.05). After successful modeling, the model group was separated into five groups: the model group (MG), the alcohol group (AG), the low-dose group (SH-L), the medium-dose group (SH-M), and the high-dose group (SH-H), plus the control group (NC), for a total of six experimental groups. The normal and model groups received a standard diet, while the alcohol group received a standard diet augmented with an equal amount of edible alcohol to Huangjiu. The low-dose, medium-dose, and high-dose groups received a conventional diet supplemented with 8.3 mL/(kg·BW), 16.6 mL/(kg·BW), and 50 mL/(kg·BW) of CSCHJ, respectively. Based on Huangjiu’s polysaccharide concentration (15.54 g/L), the low-, medium-, and high-dose groups had polysaccharide concentrations of 128.98 mg/kg, 257.96 mg/kg, and 777 mg/kg, respectively. The volume of gavage supplied was 0.1 mL/10 g body weight, and the treatment regimen lasted four weeks. The animal experiments were authorized by the Shaanxi University of Technology Animal Ethics Committee (SNUT2023106) and carried out in compliance with the rules of the Committee on Care and Use of Laboratory Animals.

### 2.3. Observation of Basic Physiological Indexes

Throughout the duration of the experiment, diligent observations were made on a daily basis to assess the morphology, activity, and fecal status of the mice. The morphology of mice mainly includes the fur color and smoothness, mental state, and response to external stimuli. The fecal status mainly includes the color and shape. Daily records were maintained regarding their food intake, while weekly body weight measurements were conducted to monitor changes over time.

### 2.4. Measurement of Fasting Blood Glucose Values

Following a 12 h fasting period, blood samples were collected from the tail of each mouse, and the fasting blood glucose (FBG) levels were measured using a blood glucose meter (EA-11, Sinocare Biosensing Co., Ltd., Changsha, China). The initial FBG values of each group of mice were recorded prior to the commencement of the experimental intervention. Subsequently, the FBG values of each group of mice were measured on the seventh day of each week throughout the intervention period.

### 2.5. Measurement of Biochemical Indexes

#### 2.5.1. Measurement of Blood Lipid Levels

After the experiment was over, the mice’s orbital veins were used to draw blood samples into 10 mL centrifuge tubes through the eye socket. After that, these samples were kept for four hours at room temperature. The blood samples were then centrifuged for 15 min at 4 °C at a rate of 4000 revolutions per minute (r/min) in order to isolate the serum. After that, the serum was kept at −20 °C for later examination. Using commercially available assay kits, the amounts of TC, TG, HDL-C, and LDL-C in the serum were measured.

#### 2.5.2. Measurement of the Index of the Liver, Kidney, and Epididymal Fat

Following anesthesia, cervical dislocation was used for execution. Then, the mice were immediately dissected, and the liver, kidneys, and epididymal fat were meticulously retrieved using tweezers. After extraction, the tissues were gently rinsed with physiological saline solution, and any excess surface moisture was removed using filter paper. The tissues were then weighed and measured. The indices of the liver, kidneys, and epididymal fat were calculated using Formula (1).

Organ and epididymal fat Index (%) = Organ and epididymal fat weight/Body weight × 100%(1)

#### 2.5.3. Measurement of the Liver and Kidney Function

Assay kits were used to measure the amounts of ALT, AST, Scr, and BUN in the serum, which were used to assess the liver and kidney function. The experimental methods were carried out in accordance with the kits’ instructions, which ensured that the necessary operating steps were followed. The dilution ratio of the samples was adjusted to meet the specific requirements of the experiment.

### 2.6. Hematoxylin-Eosin (H&E) Staining of Liver and Kidney Tissue Sections

Following euthanasia, the mice were promptly dissected, and the liver and kidneys were expeditiously isolated from the chest and abdominal cavity. Subsequently, the tissues were meticulously cleaned to remove fascia and blood. After that, the whole liver and kidneys were weighed, and the results were noted. A fraction of the liver and kidney tissues was fixed by immersion in a 4% (*w*/*v*) paraformaldehyde solution, and the remaining portion was frozen at −80 °C. After the fixation process was finished, the tissues of the liver and kidney were dehydrated in ethanol solutions and then embedded in paraffin. Following tissue sectioning into 4 μm slices, the tissues underwent H&E staining for histological analysis.

### 2.7. Measurement of SCFAs in Mice Feces

The method for detecting SCFAs in feces closely follows Feng’s protocol [19], with appropriate adjustments. Initially, 40 mg of the fecal sample was homogenized in 1 mL of saturated NaCl solution at 40 Hz for 4 min. Subsequently, the homogenized sample was thoroughly mixed with 40 μL of 10% H_2_SO_4_. Next, 800 μL of methyl tert-butyl ether with 25 mg/L 2-methylvaleric acid was added to extract SCFAs in the feces by ultrasound for 5 min. The resultant mixture was then centrifuged at 10,000 rpm for 15 min. To help with water absorption from the extraction solution, 0.25 g of anhydrous Na_2_SO_4_ was added after the upper layer of the solution was carefully collected into a centrifuge tube. After that, the mixture was centrifuged for 15 min at 10,000 rpm to extract the supernatant. Gas chromatography–mass spectrometry was utilized to determine the SCFAs present in the supernatant. An HP-FFAP column (30 m × 250 μm × 0.25 μm) was acquired from Agilent Technologies Co., Ltd., located in Santa Clara, CA, USA. A Shimadzu QP2020 NX mass spectrometer (Shimadzu (China) Co., Ltd., Shanghai, China) was used for the detection. With an injection volume of 1 μL and a flow rate of 1 mL/min, helium was utilized as the carrier gas. The temperature was raised to 80 °C and held there for 1 min. It was then raised to 200 °C at a pace of 10 °C/min and held there for 5 min. Finally, it was raised to 240 °C at a rate of 40 °C/min and held there for 1 min. And 200 °C was the ionization temperature.

### 2.8. Analysis of Gut Microbiota in Mice

After extracting DNA from mouse fecal samples, the V4 region of the 16S rDNA was amplified using the primers 515F (5′-GTGCCAGCMGCCGCGGTAA-3′) and 806R (5′-GGACTACHVGGGTWTCTAAT-3′) in a polymerase chain reaction (PCR). Following that, Illumina HiSeq 2500 (Illumina, Inc., San Diego, CA, USA) sequencing was performed on the PCR results. Upon the completion of sequencing, overlapping sequences were utilized to concatenate the paired-end data. Subsequent steps involved quality control measures and chimeric filtering to ensure the generation of high-quality clean data. DADA2 (Differential Amplicon Denoising Algorithm) no longer clusters based on sequence similarity but instead obtains representative sequences with single base accuracy through steps such as “deduplication” (equivalent to clustering based on 100% similarity), greatly improving data accuracy and species resolution. The core of DADA2 is denoising, and then the concept of ASVs (Amplicon Sequence Variants) is used to construct an Operational Taxonomic Units (OTU) table, obtaining the final ASV feature table and feature sequence for further diversity analysis, species classification annotation, and difference analysis.

### 2.9. Statistical Analysis

Data were represented as the means ± standard deviation ($\bar{x}\pm s$). The statistical analysis of data was subjected to a one-way ANOVA, followed by Dunnett’s multiple comparisons test in GraphPad Prism version 8.0. Statistical significance was defined as a *p* value < 0.05, while an extremely significant statistical value was defined as a *p* value < 0.01.

## 3. Results

### 3.1. The Main Ingredients of CSCHJ

The alcohol content, polysaccharides, total acids, and amino nitrogen in CSCHJ were determined (specific methods shown in the Supplementary Materials), and the results are shown in Table 1. All components meet the physical and chemical indicators of dry yellow wine, and the polysaccharide content is relatively high, which may make a major contribution to its physiological function.

### 3.2. Establishment of Animal Model of Hyperlipidemia

The normal control group and model group were subjected to a diet regimen comprising a basic diet and high-fat diet, respectively, for a duration of 4 weeks. Orbital blood samples were collected from the mice for the determination of TC, TG, HDL-C, and LDL-C. The results are presented in Table 2. As depicted in the table, the control group exhibited an extremely significant difference in TC compared to the model group (*p* < 0.01), while the other three indicators of hyperlipidemia displayed significant differences (*p* < 0.05). These findings collectively affirm the successful establishment of the hyperlipidemia mouse model.

### 3.3. Effect of CSCHJ on the Mouse Physiological State

**Figure 1:**
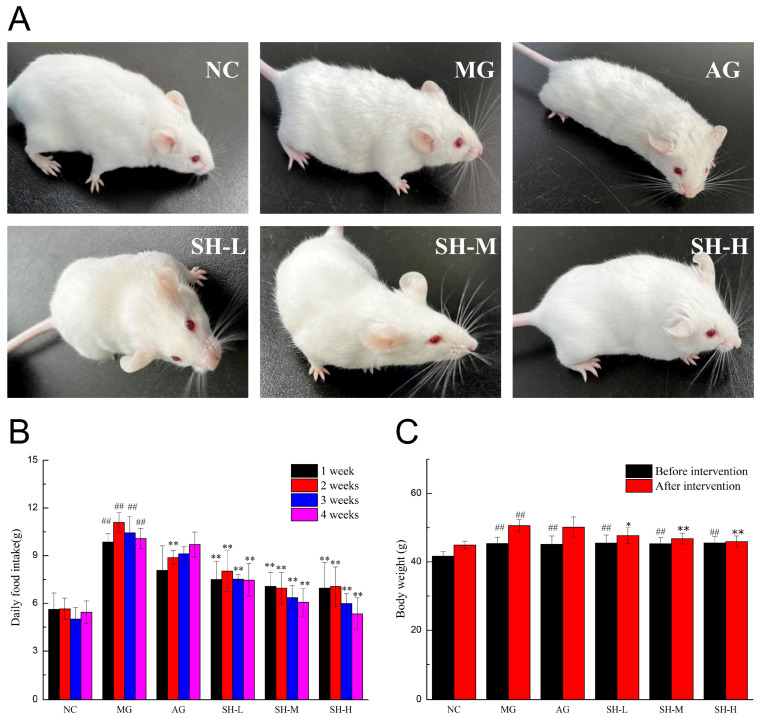
Effect of CSCHJ on mouse physiological state. (**A**) General morphology; (**B**) Daily food intake; (**C**) Body weight. NC: normal control group; MG: model group; AG: alcohol group; SH-L: *Stachys sieboldii* Miq. Huangjiu low-dose group; SH-M: *Stachys sieboldii* Miq. Huangjiu medium-dose group; SH-H: *Stachys sieboldii* Miq. Huangjiu high-dose group. ## *p* < 0.01 versus the normal control group. * *p* < 0.05, ** *p* < 0.01 versus the model group.

During the molding process, the NC group mice had a smooth fur color, a normal mental state, a red conjunctiva, a flexible response to the outside world, a good appetite and stable food intake, and feces with normal color, softness, and morphology. The overall morphology of the mice fed with a high-fat diet showed significant differences compared to the NC group. The specific situation was as follows: rough and dull fur, mental fatigue, deep red conjunctiva, increased appetite, and loose and soft stools that do not form. After 4 weeks of the experimental intervention, the AG group and MG group mice had a significantly higher food intake than the NC group and an increased response to external stimuli. The CSCHJ intervention in various dosage groups of mice showed that the overall mental state improved with the increase in the polysaccharide concentration, the reaction sensitivity increased, the activity increased, the fur color and glossiness increased, the food intake decreased compared to that of the MG group, and feces formed. The general morphology of mice in each experimental group is shown in Figure 1A. In summary, the results of CSCHJ improving the physiological state of mice preliminarily indicate CSCHJ has a dose–response relationship in inhibiting hyperlipidemia in animal models.

The daily food intake results of each group of mice before and after the intervention are shown in Figure 1B and Table S1. The daily food intake of the NC group remained stable at around 5.5 g, and there was no significant difference before and after the intervention. Compared with the NC group, the daily food intake of MG and AG showed a significant increase both before and after the intervention (*p* < 0.01). Due to the balance of energy metabolism in the mice, the daily food intake of AG was slightly lower than that of MG. The mice in the CSCHJ dose group had a significantly lower daily food intake than those in the MG group (*p* < 0.01). Additionally, there was a negative correlation between the CSCHJ dose and daily food intake, suggesting that CSCHJ regulates mice’s appetite.

The changes in the body weight of each group of mice are shown in Figure 1C. Before and after the intervention, the growth status of each group of mice was well. The body weight of the NC mice remained stable at 41 g, while that of the mice fed with a high-fat diet in each group significantly increased to 45 g (*p* < 0.01). After the intervention, changes in the body weight of each group were observed. Especially in the MG group, the body weight increased by 11.60%, showing an extremely significant difference compared to the NC group (*p* < 0.01) and indicating the presence of severe metabolic problems in the MG group. Compared with the MG group, the body weight of the AG group was slightly lower (50.16 g), which indicates that alcohol has no effect on regulating body weight. The body weight of the *Stachys sieboldii* Miq. Huangjiu group showed an increasing trend, but the growth rate was alleviated. Among them, the body weight of the SH-L group increased by 4.81%, showing a significant difference compared to the MG group (*p* < 0.05), while those of the SH-M and SH-H groups increased by 3.36% and 0.83%, respectively, showing an extremely significant difference compared to the MG group (*p* < 0.01). These results indicate that CSCHJ could effectively intervene the body weight gain of hyperlipidemic mice, and the growth rate of the body weight is negatively associated with the increase in the polysaccharide concentration. The reason may be that the mice themselves achieve a balance between energy intake and metabolism, leading to the inhibition of appetite.

### 3.4. Effect of CSCHJ on the Glucose and Lipid Metabolism of Mice

**Figure 2:**
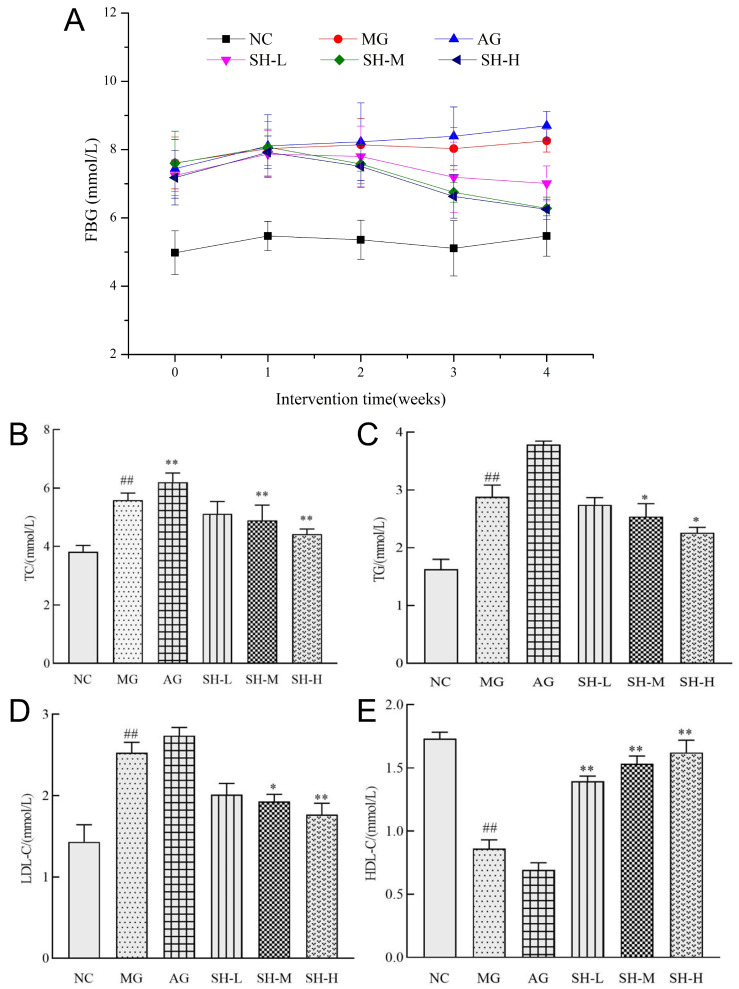
Effect of CSCHJ on the glucose and lipid metabolism of mice. (**A**) FBG; (**B**) TC; (**C**) TG; (**D**) LDC-C; (**E**) HDL-C. NC: normal control group; MG: model group; AG: alcohol group; SH-L: *Stachys sieboldii* Miq. Huangjiu low-dose group; SH-M: *Stachys sieboldii* Miq. Huangjiu medium-dose group; SH-H: *Stachys sieboldii* Miq. Huangjiu high-dose group. ## *p* < 0.01 versus the normal control group. * *p* < 0.05, ** *p* < 0.01 versus the model group.

Hyperlipidemia often coexists with other metabolic diseases due to abnormal lipid metabolism, such as abnormal glucose metabolism [20], obesity [21], etc. In this study, the FBG concentration in the MG group induced by a high-fat diet was 7.61 ± 0.76, which was significantly increased compared to that of the NC group (*p* < 0.01), and the detailed results are shown in Table S2 and Figure 2A. Before the intervention, the FBG concentrations of AG, SH-L, SH-M, and SH-H were 7.44 ± 0.86, 7.24 ± 0.46, 7.60 ± 0.94, and 7.18 ± 0.80, respectively; these were not significantly different from that of MG. After 4 weeks of the experimental intervention, the average FBG concentration of the NC group was 5.47 mmol/L, which was not significantly different from that before the intervention, indicating that the FBG concentration of normal mice will be maintained at around 5 mmol/L. The FBG concentration of the MG and AG groups reached 8.26 ± 0.33 and 8.70 ± 0.42 mmol/L and increased by 8.54% and 16.94% compared with that before the intervention, respectively. This demonstrates that alcohol has no effect on lowering blood sugar and may increase the risk of hyperglycemia. The overall FBG concentration of mice in the Huangjiu intervention group showed a downward trend with the intervention time. The SH-L group showed an extremely significant decrease (*p* < 0.01) compared to the MG group at the 4th week of intervention, with an FBG concentration of 7.01 mmol/L. The FBG concentration in both the SH-M and SH-H groups showed an extremely significant decrease (*p* < 0.01) at the 3rd week of the intervention. At the 4th week, the FBG concentration was 6.28 and 6.25 mmol/L, with decreases of 17.37% and 12.95%, respectively. The results show that CSCHJ has a certain promoting effect on reducing blood glucose levels in hyperlipidemic mice, and there is a negative correlation between the FBG concentration and polysaccharide concentration.

The main reason why CSCHJ improves the physiological status of hyperlipidemic mice is to regulate their lipid metabolism, and the results are shown in Figure 2B–E. Compared with the NC group, the MG group showed a significant increase in TC, TG, and LDL-C levels, while the HDL-C level decreased significantly, which shows obvious abnormalities in lipid metabolism in the MG group. Compared with the MG group, the AG group showed a significant increase in TC and TG levels and a certain degree of an increase and decrease in LDL-C and HDL-C levels, respectively, which indicates that pure alcohol intervention exacerbated the degree of hyperlipidemia in mice.

In contrast, compared with the MG group, the TC levels in the SH-L, SH-M, and SH-H groups of mice decreased by 8.41%, 12.52%, and 20.93%, respectively, the serum LDL-C levels decreased by 20.55%, 23.72%, and 30.04%, respectively, and the HDL-C levels increased by 61.63%, 77.91%, and 88.37%, respectively. The above three indicators in the SH-H groups showed extremely significant differences (*p* < 0.01), and in the SH-M groups, they showed significant differences (*p* < 0.05). In addition, the serum TG levels in the SH-L, SH-M, and SH-H groups decreased by 4.86%, 11.81%, and 21.53% compared to those in the MG group, respectively, with significant decreases observed in the medium- and high-dose groups (*p* < 0.05). The results indicate that intervention with polysaccharide functional factors in CSCHJ could effectively alleviate lipid metabolism abnormalities in hyperlipidemic mice and has a certain dose–response relationship.

### 3.5. Effect of CSCHJ on the Organs of Mice

**Figure 3:**
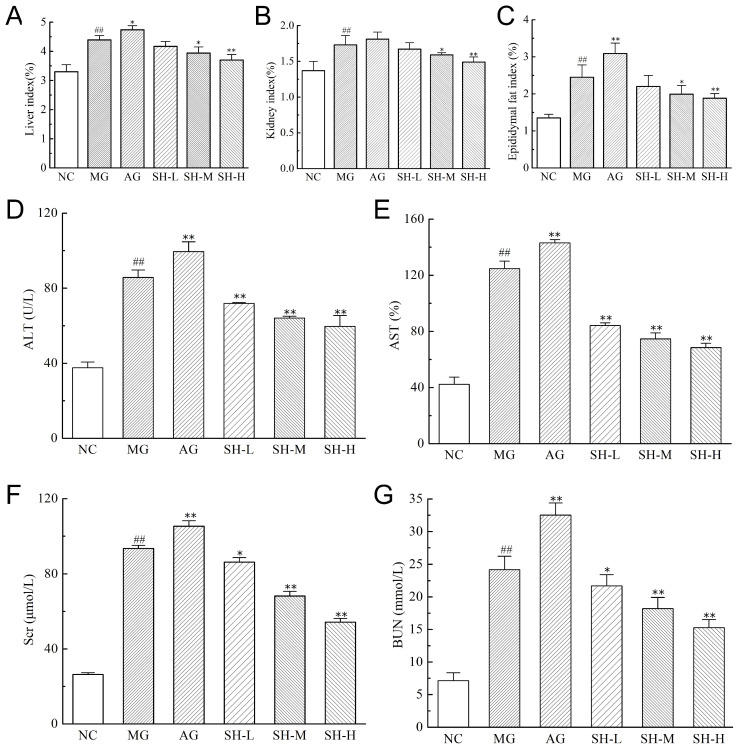
Effect of CSCHJ on the visceral organs of mice. (**A**) Liver index; (**B**) kidney index; (**C**) epididymal fat index; (**D**) ALT; (**E**) AST; (**F**) Scr; (**G**) BUN. NC: normal control group; MG: model group; AG: alcohol group; SH-L: *Stachys sieboldii* Miq. Huangjiu low-dose group; SH-M: *Stachys sieboldii* Miq. Huangjiu medium-dose group; SH-H: *Stachys sieboldii* Miq. Huangjiu high-dose group. ## *p* < 0.01 versus the normal control group. * *p* < 0.05, ** *p* < 0.01 versus the model group.

The mouse organ index is the ratio of organs to their body mass. For healthy mice, the ratio is generally relatively stable. If the organ index is higher or lower than the normal level, it can be judged that the organs have hypertrophy, hyperplasia, or pathological atrophy [22]. In this study, hyperlipidemia mice induced by a high-fat diet had abnormal metabolism due to the long-term consumption of a high-fat diet. This accelerated fat accumulation in the body, increased the liver and kidney burden, and caused a certain degree of liver and kidney damage and enlargement, which was manifested by liver, kidney, and epididymal fat index increases (Figure 3A–C).

Compared with the NC group, the liver, kidney, and epididymal fat index of MG showed an extremely significant increase, with increases of 33.03%, 26.28%, and 81.48%, respectively. Meanwhile, it was observed that the liver and kidney tissues of the MG group mice were significantly enlarged, and the accumulation of fat in the MG group mice was also increased. Compared with MG, the AG results showed that the intake of pure alcohol accelerated tissue enlargement and fat accumulation. In contrast, different doses of the CSCHJ group can effectively reduce relevant indices and alleviate the damage of hyperlipidemia to visceral organs. The SH-H group showed a significant decrease in the liver, kidney, and epididymal fat index levels (*p* < 0.01), with decreases of 15.72%, 24.00%, and 23.27%, respectively. The results indicate that CSCHJ can effectively alleviate the organ enlargement and fat accumulation caused by hyperlipidemia in mice, and it shows a significant dose–response relationship.

Aminotransferase mainly exists in liver cells, and the serum aminotransferase activity will increase when the liver tissue is damaged. In clinical practice, ALT and AST are commonly used as indicators to evaluate liver function [23]. The increased activity of ALT and AST in serum indicates certain damage to the liver tissue. According to Figure 3D,E, the ALT and AST activities in the MG group were significantly higher than those in the NC group (*p* < 0.01), indicating hyperlipidemia significantly upregulated the levels of ALT and AST and caused a certain degree of liver damage [24]. Notably, the ALT and AST activities were significantly upregulated in the MG group after pure alcohol treatment (*p* < 0.01), which suggests pure alcohol will exacerbate liver damage. In contrast, the liver damage was strikingly inhibited by CSCHJ treatment (*p* < 0.01). Compared with the MG group, the ALT activity of the SH-L, SH-M, and SH-H groups decreased by 16.13%, 25.21%, and 30.41%, respectively, while the AST activity decreased by 32.51%, 40.15%, and 54.11%, respectively. The results indicate that CSCHJ can alleviate the liver damage caused by hyperlipidemia, and it shows a significant dose–response relationship.

Hyperlipidemia is closely related to kidney injury. Lipids deposited in the kidneys can directly or indirectly affect the structure and function of the glomeruli and tubules and ultimately lead to kidney injury [25]. In clinical practice, Scr and BUN are used as important indicators to evaluate renal function, and their levels being above the normal range indicates the occurrence of kidney injury [26]. The changes in the Scr and BUN content in each group of mice are shown in Figure 3F,G. The Scr and BUN contents in the MG group were significantly higher than those in the NC group (*p* < 0.01) and increased by 2.51 times and 2.39 times, respectively, which indicates that the renal function of mice with hyperlipidemia is severely damaged. It can be expected that the intake of pure alcohol further affects lipid metabolism in the body and exacerbates kidney injury in AG mice. However, the kidney injury was markedly ameliorated by the CSCHJ through different concentrations of polysaccharides (*p* < 0.01). The higher the concentration of the CSCHJ intake, the more significant the inhibitory effect on kidney injury in hyperlipidemic mice, and the high-dose CSCHJ made the Scr and BUN content decline by 26.99% and 24.73%, respectively. The results show that CSCHJ with different polysaccharide concentrations has a positive effect on the changes in the Scr and BUN content in hyperlipidemic mice, thereby alleviating the kidney injury caused by hyperlipidemia.

**Figure 4:**
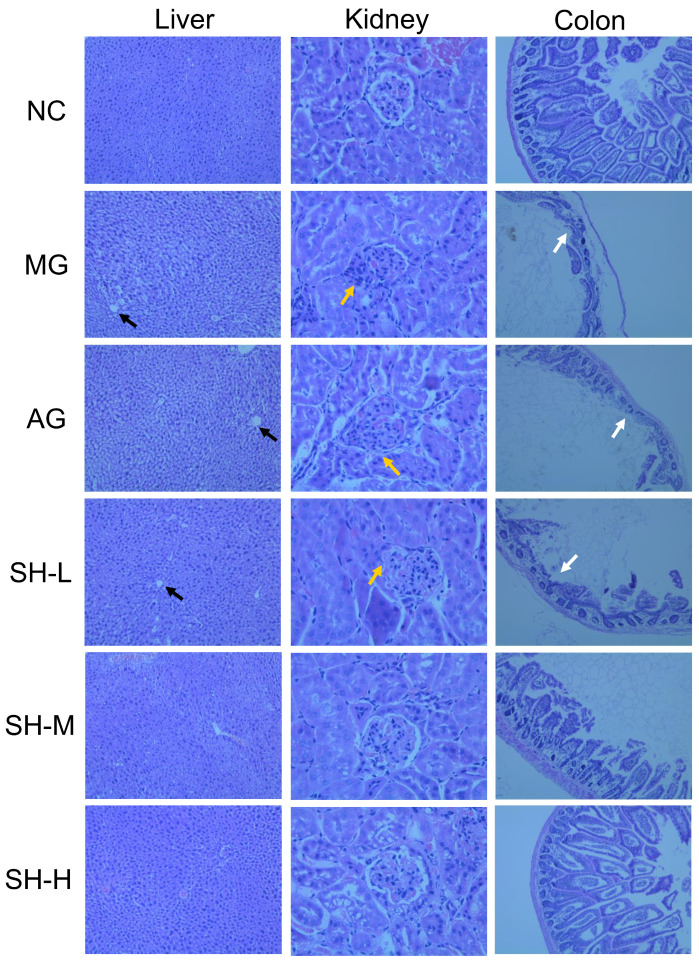
Histological staining of the liver, kidney, and colon tissue of each experimental group. NC: normal control group; MG: model group; AG: alcohol group; SH-L: *Stachys sieboldii* Miq. Huangjiu low-dose group; SH-M: *Stachys sieboldii* Miq. Huangjiu medium-dose group; SH-H: *Stachys sieboldii* Miq. Huangjiu high-dose group.

Histological staining was used to evaluate changes in liver, kidney, and colon features to further explore the alleviative effect of CSCHJ in hyperlipidemic mice, and the results are shown in Figure 4. The morphology of liver cells in the NC group was normal, with a regular arrangement, a consistent size, clear intercellular boundaries, without lesions, fat droplets, and inflammatory infiltration. However, there were large amounts of fat droplets in the liver tissue of the MG group (blank arrow in Figure 4). In addition, the disordered arrangement, uneven size, and unclear intercellular boundaries of liver cells, as well as the uneven cytoplasmic staining, indicate that the liver cell structure of this group has been disrupted. It was obvious that the intake of pure alcohol in the AG group further exacerbated liver cell damage, while CSCHJ with different polysaccharide concentrations had a positive effect on alleviating liver damage in hyperlipidemic mice. The alleviating effect increases with the increase in the polysaccharide concentration. The results showed that the degree of liver cell damage of the SH-H group was relatively mild, and the cell morphology tended to be normal, with clear boundaries and almost uniform staining. Combining the above liver index and liver function evaluation, these results further confirm the alleviating effect of CSCHJ on liver injury in hyperlipidemic mice, and it has a dose–response relationship.

As shown in Figure 4, the kidney tissue cells of the NC group mice had a clear structure, uniform morphology, and regular distribution. The glomerulus was not dilated, and the basement membrane, the volume, and the space of the renal sacs were normal. In contrast, the kidney tissue cells structure of the MG group mice was blurry, and the glomeruli showed an expanding trend (yellow arrow in Figure 4). The number of glomerular cells increased and showed a trend of expansion, while the gap between the renal sacs became smaller and some inflammatory cells were presented in the surrounding area. Overall, these results suggest that the kidney tissue cells of MG group mice may be severely damaged, while the extent of kidney tissue cellular damage is more pronounced in AG group mice. In contrast, under the intervention of CSCHJ, the kidney tissue cellular damage showed corresponding relief with the increase in the polysaccharide concentration. Combining the above kidney index and renal function evaluation, the intervention of CSCHJ can alleviate glomerular and tubular injury, inhibit peripheral inflammatory cell infiltration, restore the overall kidney tissue cell morphology, and alleviate the damage of hyperlipidemia to the kidney.

As shown in Figure 4, the glands in the colonic mucosal layer of the NC group were arranged neatly and upright, with a small gap between them and the mucosal muscle layer. The overall arrangement of the glands was uniform, and the mucosa was thick. The morphology of the colon tissue sections indicates that the colon tissue structure of the NC group mice is normal and without any lesions. However, the colon mucosal glandular shape of the MG and AG group mice showed significant atrophy (white arrow in Figure 4); it was a decrease in number or disappearance and an increase in glandular spacing. These results indicate that the colon mucosa of hyperlipidemic mice becomes thinner, and the colon tissue may suffer greater damage under pure alcohol intervention. In contrast, the glandular morphology gradually recovered as the dosage of CSCHJ increased, and the glandular morphology in the SH-H group approached that in the NC group. The results indicate that CSCHJ can alleviate the abnormal state of glandular morphology, quantity, and muscular layer status in colonic mucosal tissue caused by hyperlipidemia and has a good dose–response relationship.

### 3.6. Effect of CSCHJ on Fecal SCFAs Content in Hyperlipidemic Mice

**Figure 5:**
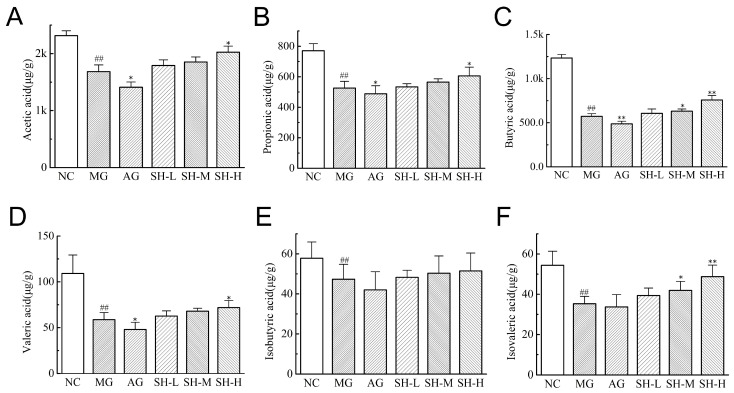
Effect of CSCHJ on fecal SCFAs content in hyperlipidemic mice. (**A**) Acetic acid; (**B**) Propionic acid; (**C**) Butyric acid; (**D**) Valeric acid; (**E**) Isobutyric acid; (**F**) Isovaleric acid. NC: normal control group; MG: model group; AG: alcohol group; SH-L: *Stachys sieboldii* Miq. Huangjiu low-dose group; SH-M: *Stachys sieboldii* Miq. Huangjiu medium-dose group; SH-H: *Stachys sieboldii* Miq. Huangjiu high-dose group. ## *p* < 0.01 versus the normal control group. * *p* < 0.05, ** *p* < 0.01 versus the model group.

The contents of SCFAs in the feces of each group mice are shown in Figure 5; the contents of acetic acid, propionic acid, and butyric acid were high, while those of valeric acid, isobutyric acid, and isovaleric acid were low. The content of all SCFAs in the MG group was significantly lower than that in the NC group (*p* < 0.01), which indicates that hyperlipidemia leads to a downregulation of SCFAs content in the intestine of mice and is consistent with the results of literature reports [27]. After 4 weeks of the intervention with alcohol, the overall SCFAs content in the AG group continued to show a downward trend compared to that of the MG group. In contrast, the contents of overall SCFAs gradually increased with the increase in the CSCHJ dosage after the intervention. The content of butyric acid and isovaleric acid in the SH-M group showed a significant increase (*p* < 0.05), up 10.51% and 17.14%, respectively. In the SH-H group, the contents of acetic acid, propionic acid, and valeric acid were increased significantly (*p* < 0.05), with increases of 20.33%, 15.02%, and 22.58%, respectively, and the increase in butyric acid and isovaleric acid was more significant (*p* < 0.01), with increases of 32.75% and 38.14%, respectively. Meanwhile, the content of isobutyric acid also increased, but there was no significant difference compared to that of the MG group. The results indicate that alcohol can downregulate the content of SCFAs in the intestinal tract of hyperlipidemic mice, while the content of SCFAs will be upregulated by the polysaccharide in CSCHJ and has a significant dose–response relationship.

### 3.7. CSCHJ Reshaped the Gut Microbial Structure in Hyperlipidemic Mice

The fecal microbiota were analyzed using 16S rDNA sequencing technology to explore the regulatory effects of CSCHJ on the gut microbiota of hyperlipidemic mice. The statistical results of the sample sequencing are presented in Table S3. A total of 1,512,071 sequences were obtained from 6 groups comprising 18 samples, of which 1,312,901 were deemed valid sequences. This yielded a ratio of valid data to raw data of 86.86%. Among the valid data, the number of sequences with data quality ≥Q20 and Q30 was 1,276,796 and 1,214,827, respectively, accounting for 97.25% and 92.53% of the total valid data. The sequence range per sample was 80,000 to 90,000, with an average of 84,003 sequences per sample. Figure S1 depicts the dilution curves of each group, which tend to flatten with increasing sequencing quantity. This observation indicates the reliability of the experimental data and suggests high species abundance. The alpha diversity index of each group is summarized in Table 3. According to the Chao1 index, it is evident that a long-term high-fat diet can suppress the diversity of mouse gut microbiota [28]. However, CSCHJ intervention appears to partially restore the diversity of gut microbiota.

**Figure 6:**
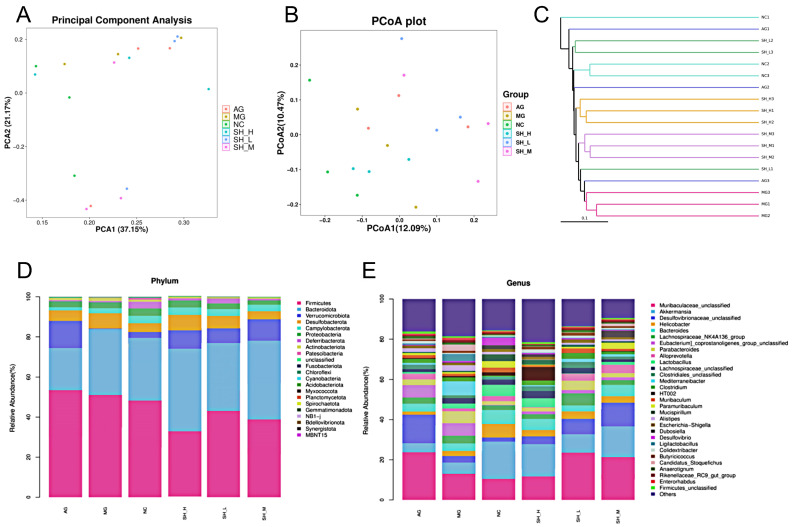
Effect of CSCHJ on the gut microbial structure in hyperlipidemic mice. (**A**) PCA analysis map; (**B**) PCoA map; (**C**) UPGMA hierarchical clustering analysis; (**D**) Changes in gut microbiota at the phylum level; (**E**) Changes in gut microbiota at the genus level.

The principal component analysis (PCA) of the gut microbial structure in six groups of samples is shown in Figure 6A. The NC group and MG group were concentrated in two different regions, which indicates that hyperlipidemia causes significant changes in the structure of the gut microbiota. In addition, the similarity between the MG group and the AG group suggests that there may be a certain connection between the microbial structures of the two groups, and alcohol does not have the function of restoring the structure of gut microbiota in hyperlipidemic mice. After the intervention by CSCHJ, the microbial structures of the SH-H group were the closest to that of the NC group, which reveals that the high-dose group of CSCHJ plays a role in restoring the gut microbiota in hyperlipidemic mice. According to the principal co-ordinates analysis (PCoA) map (Figure 6B), there were significant differences in the structure of the gut microbiota among the different groups. The NC group was similar to the SH-H group, while the MG group was similar to the AG group. The results indicate that CSCHJ is different from edible alcohol and plays a positive role in regulating the gut microbiota in hyperlipidemic mice. The unweighted pair-group method with arithmetic means (UPGMA) hierarchical clustering analysis of all groups is shown in Figure 6C. The structure of the gut microbiota in the MG group was similar to that in the AG group, and the NC group was similar to SH-H group, while there were significant differences among the other groups, indicating that CSCHJ is different from edible alcohol and plays a positive role in regulating the gut microbiota in hyperlipidemic mice. The results once again indicate that CSCHJ has the effect of restoring the structure of the gut microbiota in hyperlipidemic mice, while the edible alcohol has no such efficacy.

The phylum changes in the gut microbiota are analyzed and shown in Figure 6D. A total of 22 bacterial phyla were detected, and the dominant bacterial communities with a higher abundance in each group mainly included *Firmicutes*, *Bacteroidota*, *Verrucomicrobiota*, *Desulfobacteria*, *Deferribacteres*, *Proteobacteria*, etc. *Firmicutes* and *Bacteroidetes* are the two main bacterial communities in the gut microbiota that affect the energy metabolism balance, and the content and proportion of *Firmicutes* and *Bacteroidetes* can serve as a basis for determining the potential for obesity. Normally, the abundance of *Firmicutes* in the gut microbiota of obese individuals is upregulated, while the abundance of *Bacteroidetes* is downregulated [29]. Compared with the NC group, the abundance of *Firmicutes* in the MG group increased by 6.06%, while the abundance of *Bacteroidetes* decreased by 6.37%, which is consistent with the results of literature reports [30,31]. Notably, the abundance of *Firmicutes* in the AG group was 2.87% higher than that in the MG group, while that of *Bacteroidetes* was 27.17% lower than that in the MG group, which indicates that the intake of pure alcohol accelerates the imbalance of Firmicutes and *Bacteroidetes*, and the imbalance in their ratio may be related to the body weight and organ index of hyperlipidemic mice. In contrast, the abundance of *Firmicutes* in the SH-L, SH-M, and SH-H groups decreased by 17.14%, 25.69%, and 34.29% compared to that in the MG group, while the abundance of *Bacteroidetes* increased by 11.65%, 31.10%, and 40.81%, respectively. The results show that CSCHJ is different from edible alcohol and significantly inhibits the increase in *Firmicutes* and the decrease in *Bacteroidetes* in the intestine of hyperlipidemic mice, resulting in their abundance and proportion being close to those of the NC group. Furthermore, according to the degree of changes in the two bacteria, it could be inferred that the regulatory effect becomes more pronounced with the increase in the polysaccharide concentration, which indicates that polysaccharides in CSCHJ have a positive regulatory effect on the gut microbiota of hyperlipidemic mice.

The differences in gut microbiota at the genus level are further analyzed and shown in Figure 6E. The relatively abundant bacterial communities in the six groups of mice mainly included *Muribaculaceae*, *Akkermansia*, *Desulfovibrionaceae*, *Helicobacter*, *Bacteroides*, *Lactobacillus*, *Alloprevotella*, *Paraacteroides*, *Clostridium*, etc. Among them, *Bacteroides*, *Akkermansia*, and *Lactobacillus* were the dominant genera. The relative abundance of *Bacteroides*, *Akkermansia*, and *Lactobacillus* in the MG group was lower than that in the NC group. This decline suggests a downregulation of probiotic abundance in the gut of hyperlipidemic mice induced by a high-fat diet, correlating with a decrease in overall gut health. Furthermore, compared to the MG group, the proportion of *Bacteroidetes* and *Akkermansia* in the AG group exhibited a further reduction, indicating that alcohol impedes the growth of probiotics. Conversely, the relative abundance of *Bacteroidetes* and *Akkermansia* gradually increased with escalating polysaccharide concentrations in the SH-L, SH-M, and SH-H groups. Notably, the abundance of *Lactobacillus* in the SH-H group exhibited a significant upregulation, reaching levels in the NC group. These findings suggest that CSCHJ facilitates the proliferation of probiotics in the gut of hyperlipidemic mice, thereby serving as a primary mechanism underlying the alleviation of hyperlipidemia by CSCHJ [32,33].

### 3.8. Correlation Analysis between HLP-Related Host Parameters and Gut Microbial Genera

**Figure 7:**
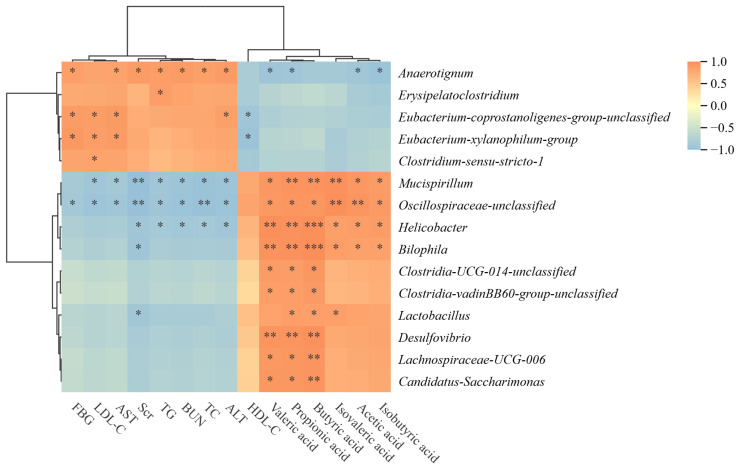
Correlation analysis heat map of microbial communities and HLP-related parameters in six groups of experimental animals. * denotes a significant correlation at the 0.05 level; ** denotes a significant correlation at the 0.01 level; *** denotes a significant correlation at the 0.001 level.

To illustrate the relationship between gut microbiota and HLP-related host parameters, the top 100 most abundant gut microbiota at the genus level of the six groups of experimental animals were correlated with their HLP-related parameters (Figure 7). The genera of *Clostridia-UCG-014-unclassified*, *Clostridia-vadinBB60-group-unclassified*, *Desulfovibrio*, *Lachnospiraceae-UCG-006*, and *Candidatus-Saccharimonas* exhibited a pronouncedly positive association with butyric acid, propionic acid, and valeric acid, with no significant relationship with HLP-related parameters, indicating that their growth increases the levels of several SCFAs but has a weak effect on alleviating hyperlipidemia. In contrast, *Erysipelatoclostridium*, *Eubacterium-coprostanoligenes-group unclassified, Eubacterium-xylanophilum-group*, and *Clostridium-sensu-stricto-1* have no significant relation with SCFAs, but they have a significant positive correlation with some parameters related to hyperlipidemia, indicating that they can directly alleviate hyperlipidemia without passing through SCFAs. However, their impact on hyperlipidemia is not very prominent. Notably, *Mucispirillum*, *Oscillospiraceae-unclassified*, and *Helicobacter* showed a pronouncedly positive relationship with all SCFAs in our investigation, while these genera presented a strikingly negative connection with almost all HLP-related parameters, indicating that they play the greatest role in alleviating hyperlipidemia.

## 4. Discussion

**Figure 8:**
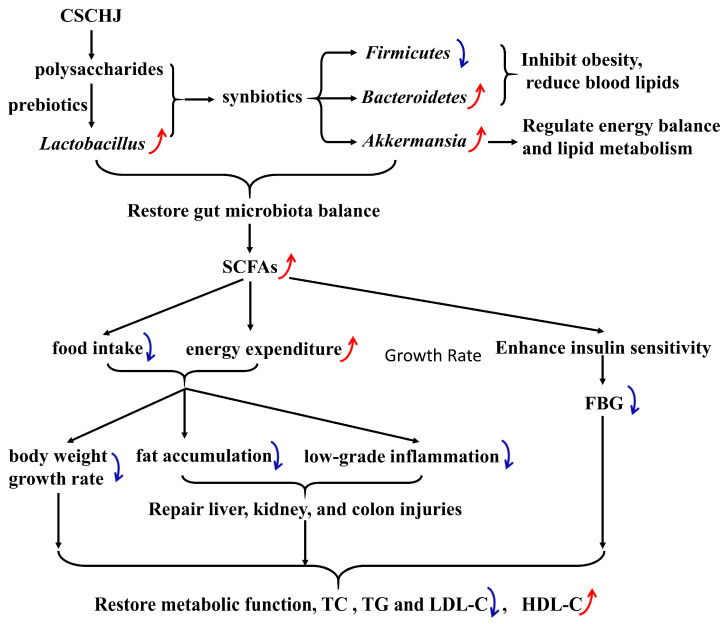
Summary of the mechanism of CSCHJ’s attenuation of hyperlipidemia. The red arrow indicates upregulation, while the blue arrow indicates downregulation.

This study assessed CSCHJ’s ability to decrease lipids using a high-fat diet-induced hyperlipidemia mouse model. CSCHJ significantly reduced the body weight and food consumption of the model mice while also improving their physiological condition. This suggests that following the CSCHJ intervention, the hyperlipidemia model mice’s energy metabolism returned to normal. In addition, the effect of CSCHJ on the four blood lipid indicators (TC, TG, LDL-C, and HDL-C) fully demonstrates its lipid-lowering effect. Given that alcohol has been shown to have no effect on blood lipid levels, CSCHJ’s high polysaccharide content (15.54 g/L) may be a significant contributing factor. Various polysaccharides have been proven to have the effect of alleviating hyperlipidemia, such as dietary ginger polysaccharides [34], *Lysimachia christinae* polysaccharide [35], *Lycium barbarum* polysaccharide [36], *grass* polysaccharides [37], etc. The main mechanisms of polysaccharides in lowering blood lipids were altering the structure of the gut microbial community, relieving liver function injury, and reducing systemic inflammation and the oxidative stress level, thus improving the metabolic outcomes and regulating dyslipidemia. A summary of the mechanism of CSCHJ’s attenuation of hyperlipidemia is shown in Figure 8.

*Stachys sieboldii* (*S. sieboldii*) Miq. is an herbaceous plant from the Labiatae family that is commonly used as a vegetable and a Chinese herbal medicine and has good therapeutic effects on ischemic brain injury, dementia, and various gastrointestinal diseases. Several studies reported that there are many bioactive polysaccharides from *S. sieboldii* Miq. Yin et al. isolated stachyose in *S. sieboldii* Miq. [38], which was regarded as a prebiotic and can decrease the *Escherichia-Shigella* genera and increase the relative abundance of the *Bifidobacteria*, *Lactobacillus*, *Faecalibacterium*, *Prevotella*, and *Bacteroides* genera [39,40]. Additionally, a different study revealed that adding *S. sieboldii* Miq. to mice’s diet enhanced beneficial gut microbiota (*Ruminococcaceae* and *Akkermansia muciniphila*) and reduced harmful microflora (*Enterobacteriaceae*) [41]. The prebiotics inside the *S. sieboldii* Miq. will be released into Huangjiu through fermentation, promoting CSCHJ containing prebiotic ingredients. The experiment showed that after intervention with CSCHJ, there were significant changes in the gut microbiota in hyperlipidemic mice, and the richness and uniformity of the microbiota were improved.

Under the action of prebiotics in Huangjiu, the abundance of *Lactobacillus* in the gut of hyperlipidemic mice is significantly increased, which can inhibit pathogenic bacteria, enhance the structure of intestinal microbiota, preserve normal intestinal function, and regulate host metabolism [42]. The prebiotics in Huangjiu and *Lactobacilli* can form synbiotics in the intestine, which will be more effective in regulating gut microbiota disorders. The combination of stachyose and *L. rhamnosus* GG could more effectively regulate gut microbiota disorders than stachyose or *L. rhamnosus* GG alone [43]. The synbiotics significantly increased the relative abundance of *Rikenella*, *Bacteroides*, *Odoribacter*, *Ruminiclostridium_5*, and *Gordonibacter* and reduced the ratio of *Firmicutes* to *Bacteroidetes*. The synbiotics composed of *Lacticaseibacillus paracasei K56* and galacto-oligosaccharide significantly upregulated the relative abundance of *Bacteroidota*, *Bifidobacterium*, *Lactobacillus*, *Faecalibacterium*, and *Blautia* and downregulated the ratio of *Firmicutes* to *Bacteroidetes* and the relative abundance of *Escherichia*-*Shigella* [44]. In actuality, the synbiotics, which included *Lactobacillus* and polysaccharides, dramatically decreased the proportion of *Firmicutes*, raised those of *Bacteroidetes* and *Akkermansia*, and downregulated the *Firmicutes*-to-*Bacteroidetes* ratio. Two of the most prevalent prokaryote species in humans, *Firmicutes* and *Bacteroidetes*, have an impact on the equilibrium of energy metabolism. Previous research has found that an elevated *Firmicutes*/*Bacteroidetes* ratio is associated with obesity [29]. It is worth noting that recent research has revealed that lowering the *Firmicutes*/*Bacteroidetes* ratio also helps to reduce blood lipids. For example, tomato seed oil [45], grain [46], Agaricus blazei Murrill polysaccharides [47], germinated brown rice [30], and Chenopodium quinoa Willd. husk saponins [31] with therapeutic promise against hyperlipidemia can all reduce the *Firmicutes*/*Bacteroidetes* ratio. In addition, numerous studies have revealed that *Bacteroides* possesses probiotic properties, primarily in its ability to assist the host in decomposing polysaccharides, accelerating intestinal mucosal angiogenesis, improving host immunity, and maintaining the intestinal microbiota balance [48,49]. *Akkermansia* is a type of mucin-degrading bacteria found in the colon that can regulate host energy balance and lipid metabolism levels as well as have a good influence on the treatment of metabolic-related illnesses. Their composition ratio is positively correlated with host health [50,51]. The relative abundance of *Bacteroides*, *Akkermansia*, and *Lactobacillus* was decreased in the MG group, indicating gut microbiota disorders and aberrant lipid metabolism. However, CSCHJ can increase the quantity of these probiotics, which could be one of the mechanisms for regulating lipid metabolism and alleviating hyperlipidemia.

Furthermore, the generation of SCFAs is influenced by gut microbiota. Out of all the SCFAs, acetic acid, propionic acid, and butyric acid make up about 90%. While some bacterial species are capable of producing propionic acid and butyric acid, the majority of gut microbiota can create acetic acid. *Bacteroides* and *Veillonella*, two members of the *Bacteroidetes* and *Firmicutes* phylum, are the primary producers of propionic acid, and *Akkermansia muciniphila* is also capable of producing it [52]. Butyric acid is mainly produced by specific bacteria in *Firmicutes*, such as *Anaerostipes* and *Faecalibacterium praussnitzii* [53,54,55]. Some bacteria in *Firmicutes* can also produce both propionic acid and butyric acid simultaneously [56]. The types and content of SCFAs are closely related to the gut microbiota [57]. So, the upregulation of *Bacteroides*, *Akkermansia*, and *Lactobacillus* by CSCHJ has a positive impact on increasing the content of SCFAs. In fact, the contents of acetic acid, propionic acid, butyric acid, valeric acid, and isovaleric acid were increased significantly in the SH-H group. Numerous investigations have proven SCFAs’ functional activity, which includes improving the intestinal barrier, maintaining the intestinal environmental balance, and regulating energy metabolism, as well as anti-inflammatory and anti-tumor properties [52].

The long-term consumption of high-fat diets can change the gut microbiota’s general structure, which can cause inflammation and insulin resistance [58,59]. Furthermore, a high-fat diet’s imbalance in the synthesis of fat factors contributes to persistent low-grade inflammation, which is linked to the emergence of a number of illnesses, including cancer, diabetes, and cardiovascular disease [60]. Normally, hyperlipidemia causes damage to mice’s liver, kidneys, and colon tissues [61], affecting normal metabolism and exacerbating hyperlipidemia [62,63,64]. The liver, kidney, and colon damage caused by a high-fat diet in this study was due to fat accumulation and inflammation in mice. SCFAs can alleviate fat accumulation and chronic low-grade inflammation by controlling the food intake and increasing lipid metabolism. Acetate is an SCFA that circulates at the highest concentrations and can be used as an energy source by the brain [65,66]. Frost et al. confirmed that colonic acetate can cross the blood–brain barrier, be absorbed by the brain, and reduce the food intake [67]. Meanwhile, Frost et al. observed that acetate in the hypothalamus could control specific neurotransmitter release through the glutamate–glutamate cycle and then inhibit appetite [67]. Furthermore, via G protein-coupled receptors 43 (GPR43), SCFAs have been shown in multiple studies to induce the release of anorectic gut hormones (glucagon-like peptide 1 and peptide YY), which in turn reduces appetite [68,69]. There are also many reports on SCFAs promoting energy expenditures. The expression of GPR43 and G protein-coupled receptors 41 (GPR41) in adipose tissue was increased, the hydrolysis of triglycerides and the oxidation of free fatty acids in adipose tissue were promoted, and body weight was decreased when SCFAs, such as acetate, propionate, butyrate, or their mixtures, were added to the diet of high-fat diet-fed mouse models [70]. Den Besten et al. found that adding either sodium acetate, sodium propionate, or sodium butyrate into high-fat diet mouse models after 10 weeks enhanced energy expenditure during both 12 h day and night periods [71]. Sahuri-Arisoylu et al. reported that the intraperitoneal injection of nanoparticle-derived acetate in high-fat diet mouse models for 6 weeks increased energy expenditure [72]. Mechanistically, the positive effect of SCFAs on energy expenditure may be due to the stimulation of GPR43 and GPR41; they have stimulatory effects on energy expenditure, as knocking out these receptors reduces the energy expenditure rate in rodents [73,74]. In this study, the decrease in food intake and body weight in mice after intervention with CSCHJ may be a comprehensive manifestation of SCFAs inhibiting appetite and promoting energy expenditure. And liver, kidney, and colon injuries in mice have been restored, which may be a comprehensive result of SCFAs inhibiting fat accumulation and chronic low-grade inflammation [60,75].

Intestinal gluconeogenesis (IGN) has beneficial effects on glucose and energy homeostasis, promotes metabolism, and regulates body weight and blood sugar levels. SCFAs prevent abnormal glucose metabolism in mice by promoting the production of IGN [76,77]. In addition, an increasing number of studies indicate that SCFAs play an important role in restoring insulin sensitivity [78]. Gao et al. reported that supplementing high-fat diet mice with butyrate could increase the expression of PPAR-γ coactivator-1 α (PGC-1 α), activate AMPK and p38, and enhance insulin sensitivity [79]. Christiansen et al. discovered that acetate and butyrate triggered the expression of GPR43 and GPR41 on the surface of rat intestinal cells, encouraging the release of insulin, peptide YY, and glucagon-like peptide 1. These actions therefore controlled the metabolism of blood lipid energy and decreased peripheral blood glucose levels [80]. In the study, the FBG levels in hyperlipidemic mice were decreased after intervention by CSCHJ, which may be a manifestation of SCFAs activating IGN, enhancing insulin sensitivity, and promoting glucose metabolism.

T2D, obesity, non-alcoholic fatty liver disease (NAFLD), and hyperlipidemia are among the prominent metabolic illnesses that are thought to be primarily caused by microbial dysbiosis, and the gut microbiota and SCFAs have an impact on the metabolism of humans [81]. The overall mechanism of using CSCHJ to alleviate hyperlipidemia in this study may be utilizing the prebiotic effect of polysaccharides, upregulating the relative abundance of probiotics, forming synbiotics, improving the gut microbiota imbalance, and then promoting the generation of postbiotics (such as SCFAs), further improving metabolic function in the body. Probiotics, prebiotics, synbiotics, and postbiotics are powerful regulators of gut microbiota, thus possessing prospects for preventing metabolic diseases [82].

## 5. Conclusions

In summary, this study revealed the potential alleviating effects of CSCHJ on hyperlipidemia in mice induced by a high-fat diet, including the inhibition of appetite, reduction in body weight and blood sugar gain, decrease in serum levels of TC, TG, and LDL-C, and elevation of HDL-C levels. Meanwhile, it was found that the alcohol in Huangjiu has an aggravating effect on the symptoms of hyperlipidemia and related physiological indicators. Therefore, the observed alleviating effect of CSCHJ on hyperlipidemia is likely attributed predominantly to its polysaccharide components. By studying the effects of CSCHJ on the gut microbiota structure, fecal SCFAs content, as well as liver, kidney, and colon damage in mice with hyperlipidemia, it is inferred that the mechanism of CSCHJ alleviating hyperlipidemia may use polysaccharides as prebiotics to upregulate the relative abundance of probiotics. The two then form synbiotics to improve the imbalance of gut microbiota, thereby promoting the production of postbiotics (such as SCFAs), further repairing liver, kidney, and colon injuries, and ultimately improving the metabolic function of the body. Current research shows that CSCHJ contains active ingredients that can alleviate hyperlipidemia, providing a theoretical basis for dietary therapy for treating hyperlipidemia. The lipid-lowering active ingredients extracted from it have broad prospects for application in the development of biomedicine and functional foods.

## Figures and Tables

**Table 1:** The main ingredients in CSCHJ.

Ingredients	Alcohol (20 °C, %)	Polysaccharides (g/L)	Total Acids (g/L)	Amino Nitrogen (g/L)
Content	17.56 ± 0.44	15.54 ± 1.30	3.65 ± 0.28	0.34 ± 0.01

**Table 2:** Detection results of blood lipid indicators in two groups of mice after modeling.

Groups	TC (mmol/L)	TG (mmol/L)	LDL-C (mmol/L)	HDL-C (mmol/L)
Normal control group	2.82 ± 0.22	1.13 ± 0.35	1.08 ± 0.38	1.69 ± 0.28
Model group	4.40 ± 0.48 **	2.24 ± 0.31 *	2.02 ± 0.14 *	1.96 ± 0.22 *

Note: TC: total cholesterol; TG: triglycerides, HDL-C: high-density lipoprotein cholesterol; LDL-C: low-density lipoprotein cholesterol. * *p* < 0.05, ** *p* < 0.01 versus the model group.

**Table 3:** Alpha diversity indices of different experimental groups.

Groups	Observed Otus	Shannon	Simpson	Chao1	Goods Coverage	Pielou
NC	828.67	6.68	0.97	829.61	1.00	0.72
MG	629.67	6.67	0.95	630.66	1.00	0.69
AG	655.00	6.67	0.73	755.51	1.00	0.70
SH-L	732.33	6.38	0.94	712.56	1.00	0.69
SH-M	762.33	6.66	0.94	732.73	1.00	0.69
SH-H	764.67	6.80	0.96	765.47	1.00	0.70

Note: NC: normal control group; MG: model group; AG: alcohol group; SH-L: *Stachys sieboldii* Miq. Huangjiu low-dose group; SH-M: *Stachys sieboldii* Miq. Huangjiu medium-dose group; SH-H: *Stachys sieboldii* Miq. Huangjiu high-dose group.

## Data Availability

The original contributions presented in the study are included in the article/Supplementary Materials; further inquiries can be directed to the corresponding author.

## Supplementary Materials

The following supporting information can be downloaded at: https://www.mdpi.com/article/10.3390/foods13152360/s1, Determination of the main ingredients in CSCHJ; Table S1: Comparison of the daily food intake of mice in each group before and after intervention; Table S2: FBG concentration of mice in each group before and after intervention; Table S3: Sample sequencing quantity statistics; Figure S1: Alpha diversity dilution curve. Refs. [83,84] are cited in Supplementary Material.

## References

[1] Zhong H., Chen K., Feng M., Shao W., Wu J., Chen K., Liang T., Liu C., Chen K. (2018). Genipin alleviates high-fat diet-induced hyperlipidemia and hepatic lipid accumulation in mice via miR-142a-5p/SREBP-1c axis. FEBS J..

[2] Caus M., Eritja À., Bozic M. (2021). Role of microRNAs in Obesity-Related Kidney Disease. Int. J. Mol. Sci..

[3] Ferguson D., Finck B.N. (2021). Emerging therapeutic approaches for the treatment of NAFLD and type 2 diabetes mellitus. Nat. Rev. Endocrinol..

[4] Chaulin A. (2023). Cardiotoxicity as a possible side effect of statins. Rev. Cardiovasc. Med..

[5] Yamashita S., Masuda D., Matsuzawa Y. (2020). Pemafibrate, a new selective pparα modulator: Drug concept and its clinical applications for dyslipidemia and metabolic diseases. Curr. Atheroscler. Rep..

[6] Fan X., Han J., Zhang F., Chen W. (2023). Red yeast rice: A functional food used to reduce hyperlipidemia. Food Rev. Int..

[7] Yang Y., Zhou Z., Liu Y., Xu X., Xu Y., Zhou W., Chen S., Mao J. (2022). Non-alcoholic components in Huangjiu as potential factors regulating the intestinal barrier and gut microbiota in mouse model of alcoholic liver injury. Foods.

[8] Wang J., Zhang B., Wu Q., Jiang X., Liu H., Wang C., Huang M., Wu J., Zhang J., Yu Y. (2022). Sensomics-assisted flavor decoding of coarse cereal Huangjiu. Food Chem..

[9] Ye Y., Wang L., Zhan P., Tian H., Liu J. (2022). Characterization of the aroma compounds of Millet Huangjiu at different fermentation stages. Food Chem..

[10] Yu H., Xie T., Xie J., Ai L., Tian H. (2019). Characterization of key aroma compounds in Chinese rice wine using gas chromatography-mass spectrometry and gas chromatography-olfactometry. Food Chem..

[11] Jin Z., Cai G., Wu C., Hu Z., Xu X., Xie G., Wu D., Lu J. (2021). Profiling the key metabolites produced during the modern brewing process of Chinese traditional rice wine. Food Res. Int..

[12] Liu R., Fu Z., Zhang F., Mao Q., Luan C., Han X., Xue J., Wang D., Qin S., Hao F. (2020). Effect of yellow rice wine on anti-aging ability in aged mice induced by d-galactose. Food Sci. Hum. Well..

[13] Yang Y., Ren Q., Zhou Z., Li X., Ren D., Ji Z., Mao J. (2024). Structural elucidation of a highly branched α-d-glucan from Huangjiu and its hepatoprotective activity via gut microbiome regulation and intestinal barrier repairment. Carbohyd. Polym..

[14] Peng L., Liu S., Ji Z., Chen S., Mao J. (2019). Structure characterisation of polysaccharide isolated from Huangjiu and its anti-inflammatory activity through MAPK signaling. Int. J. Food Sci. Technol..

[15] Chen G., Huang Z., Wu L., Wu Q., Guo W., Zhao W., Liu B., Zhang W., Rao P., Lv X. (2021). Microbial diversity and flavor of Chinese rice wine (Huangjiu): An overview of currentresearch and future prospects. Curr. Opin. Food Sci..

[16] Ren Q., Sun L., Wu H., Wang Y., Wang Z., Zheng F., Lu X., Xu J. (2019). The Changes of microbial community and flavor compound in the fermentation process of Chinese rice wine using *Fagopyrum tataricum* grain as feedstock. Sci. Rep..

[17] Tian S., Zeng W., Fang F., Zhou J., Du G. (2022). The microbiome of Chinese rice wine (Huangjiu). Curr. Res. Food Sci..

[18] Shen C., Mao J., Chen Y., Meng X., Ji Z. (2015). Extraction optimization of polysaccharides from Chinese rice wine from the Shaoxing region and evaluation of its immunity activities. J. Sci. Food Agric..

[19] Feng X., Shi Y., Zhou Z., Ji Z., Zhou W., Chen S., Mao J. (2023). Alleviation of loperamide-induced constipation with sticky rice fermented huangjiu by the regulation of serum neurotransmitters and gut microbiota. J. Sci. Food Agric..

[20] Jackson S., Creo A., Kumar S. (2022). Are clinicians aggressive enough in treating diabetes-related hyperlipidemia in youth?. Curr. Atheroscler. Rep..

[21] Song H., Shen X., Zhou Y., Zheng X. (2021). Black rice anthocyanins alleviate hyperlipidemia, liver steatosis and insulin resistance by regulating lipid metabolism and gut microbiota in obese mice. Food Funct..

[22] Shi Y., Feng R., Mao J., Liu S., Zhou Z., Ji Z., Chen S., Mao J. (2021). Structural characterization of peptides from huangjiu and their regulation of hepatic steatosis and gut microbiota dysbiosis in hyperlipidemia mice. Front. Pharmacol..

[23] Danjuma M.I., Sajid J., Fatima H., Elzouki A.-N. (2019). Novel biomarkers for potential risk stratification of drug induced liver injury (DILI): A narrative perspective on current trends. Medicine.

[24] Lu S., Yuan Y., Chen F., Zheng Y., Li C., Cao J., Xia G., Liu Z., Shen X., He Y. (2022). *Holothuria leucospilota* polysaccharides alleviate hyperlipidemia via alteration of lipid metabolism and inflammation-related gene expression. J. Food Biochem..

[25] Li W., Zhang H., Zhang L., Zhang T., Ding H. (2022). Effect of thymoquinone on renal damage induced by hyperlipidemia in LDL receptor-deficient (LDL-R-/-) mice. BioMed Res. Int..

[26] Zeng Y., Li J., Wei X., Ma S., Wang Q., Qi Z., Duan Z., Tan L., Tang H. (2023). Preclinical evidence of reno-protective effect of quercetin on acute kidney injury: A meta-analysis of animal studies. Front. Pharmacol..

[27] Wang Z., Yao W., Sun Y., Han Y., Chen X., Gong P., Zhai P., Pei S., Xie J., Ba Q. (2023). Eucommia bark/leaf extract improves lipid metabolism disorders by affecting intestinal microbiota and microbiome–host interaction in HFD mice. J. Agric. Food Chem..

[28] Tong A., Wu W., Chen Z., Wen J., Jia R., Liu B., Cao H., Zhao C. (2023). Modulation of gut microbiota and lipid metabolism in rats fed high-fat diets by Ganoderma lucidum triterpenoids. Curr. Res. Food Sci..

[29] Magne F., Gotteland M., Gauthier L., Zazueta A., Pesoa S., Navarrete P., Balamurugan R. (2020). The Firmicutes/Bacteroidetes ratio: A relevant marker of gut dysbiosis in obese patients?. Nutrients.

[30] Ren C., Zhang S., Hong B., Guan L., Huang W., Feng J., Sha D., Yuan D., Li B., Ji N. (2023). Germinated brown rice relieves hyperlipidemia by alleviating gut microbiota dysbiosis. J. Integr. Agric..

[31] Yu Y., Ji X., Song L., Cao Y., Feng J., Zhang R., Tao F., Zhang F., Xue P. (2024). Saponins from Chenopodium quinoa Willd. husks alleviated high-fat-diet-induced hyperlipidemia via modulating the gut microbiota and multiple metabolic pathways. J. Sci. Food Agric..

[32] Liang Z., He Y., Wei D., Fu P., Li Y., Wang H., Yang D., Hou X. (2024). Tree peony seed oil alleviates hyperlipidemia and hyperglycemia by modulating gut microbiota and metabolites in high-fat diet mice. Food Sci. Nutr..

[33] Wang J., Liu A., Li A., Song H., Luo P., Zhan M., Zhou X., Chen L., Zhang J., Wang R. (2023). Lactobacillus fermentum CKCC1858 alleviates hyperlipidemia in golden hamsters on a high-fat diet via modulating gut microbiota. Food Funct..

[34] Wu Q., Luo L., Luo Q., Xu L., Ma Q., Hong T., Liu L., Liu Z. (2023). Dietary ginger polysaccharides (Gps) improve symptoms in hyperlipidemia rats via alterations in gut microbiota. Heliyon.

[35] Zhou Y., Nie J., Shi C., Zheng W., Ning K., Kang J., Sun J., Cong X., Xie Q., Xiang H. (2023). *Lysimachia christinae* polysaccharide attenuates diet-induced hyperlipidemia via modulating gut microbes-mediated FXR-FGF15 signaling pathway. Int. J. Biol. Macromol..

[36] Liang J., Li X., Lei W., Tan P., Han M., Li H., Yue T., Wang Z., Gao Z. (2023). Serum metabolomics combined with 16S rRNA sequencing to reveal the effects of *Lycium barbarum* polysaccharide on host metabolism and gut microbiota. Food Res. Int..

[37] Yan J., Chen T., Li L., Liu F., Liu X., Li L. (2023). The anti-hyperlipidemic effect and underlying mechanisms of barley (*Hordeum vulgare* L.) grass polysaccharides in mice induced by a high-fat diet. Food Funct..

[38] Yin J., Yang G., Wang S., Chen Y. (2006). Purification and determination of stachyose in Chinese artichoke (*Stachys sieboldii* Miq.) by high-performance liquid chromatography with evaporative light scattering detection. Talanta.

[39] Zhao Z., Liu W., Pi X. (2021). In Vitro Effects of Stachyose on the Human Gut Microbiota. Starch-Starke.

[40] He N., Wang H., Yu S., Chen K., Wu Z., Lin X., Xiao L., Zou Y., Li S. (2024). Stachyose ameliorates obesity-related metabolic syndrome via improving intestinal barrier function and remodeling gut microbiota. J. Funct. Foods.

[41] Na E., Moon K.H., Lim S.Y. (2021). The effect of *Stachy sieboldii* miq. supplementation on modulating gut microflora and cytokine expression in mice. Comb. Chem. High Throughput Scr..

[42] Dempsey E., Corr S.C. (2022). *Lactobacillus* spp. for gastrointestinal health: Current and future perspectives. Front. Immunol..

[43] Ren D., Ding M., Su J., Ye J., He X., Zhang Y., Shang X. (2024). Stachyose in combination with *L. rhamnosus* GG ameliorates acute hypobaric hypoxia-induced intestinal barrier dysfunction through alleviating inflammatory response and oxidative stress. Free Radical Bio. Med..

[44] Zhang Q., Zhao W., He J., He J., Shi S., Sun M., Niu X., Zeng Z., Zhao Y., Zhang Y. (2024). Effect of *Lacticaseibacillus paracasei* K56 with galactooligosaccharide synbiotics on obese individuals: An in vitro fermentation model. J. Sci. Food Agric..

[45] He W.S., Li L., Rui J., Li J., Sun Y., Cui D., Xu B. (2020). Tomato seed oil attenuates hyperlipidemia and modulates gut microbiota in C57BL/6J mice. Food Funct..

[46] Deng N., He Z., Guo R., Zheng B., Li T., Liu R.H. (2020). Highland barley whole grain (*Hordeum vulgare* L.) ameliorates hyperlipidemia by modulating cecal microbiota, miRNAs, and AMPK pathways in leptin receptor-deficient db/db mice. J. Agric. Food Chem..

[47] Li Y., Lu X., Li X., Guo X., Sheng Y., Xu G., Han X., An L., Du P. (2020). Effects of *Agaricus blazei* Murrill polysaccharides on hyperlipidemic rats by regulation of intestinal microflora. Food Sci. Nutr..

[48] Zafara H., Saier M.H. (2021). Gut bacteroides species in health and disease. Gut Microbes..

[49] Ye M., Yu J., Shi X., Zhu J., Gao X., Liu W. (2021). Polysaccharides catabolism by the human gut bacterium-*Bacteroides thetaiotaomicron*: Advances and perspectives. Crit. Rev. Food Sci..

[50] Niu H., Zhou M., Zogona D., Xing Z., Wu T., Chen R., Cui D., Liang F., Xu X. (2024). *Akkermansia muciniphila*: A potential candidate for ameliorating metabolic diseases. Front. Immunol..

[51] Zhao Q., Yu J., Hao Y., Zhou H., Hu Y., Zhang C., Zheng H., Wang X., Zeng F., Hu J. (2023). *Akkermansia muciniphila* plays critical roles in host health. Crit. Rev. Microbiol..

[52] Zhang D., Jian Y., Zhang Y., Li Y., Gu L., Sun H., Liu M., Zhou H., Wang Y., Xu Z. (2023). Short-chain fatty acids in diseases. Cell Commun. Signal..

[53] Hoch T., Pischetsrieder M., Hess A. (2014). Snack food intake in ad libitum fed rats is triggered by the combination of fat and carbohydrates. Front. Psychol..

[54] Wilkinson K.R., Tucker L.A., Davidson L.E., Bailey B.W. (2021). Milk-fat intake and differences in abdominal adiposity and bmi: Evidence based on 13,544 randomly-selected adults. Nutrients.

[55] Firat Y.Y., Inanc N., Soylu M., Basmisirli E., Capar A.G., Aykemat Y. (2022). Relationship between dairy consumption and abdominal obesity. J. Am. Nutr. Assoc..

[56] Kim D., Kim J. (2017). Dairy consumption is associated with a lower incidence of the metabolic syndrome in middle-aged and older Korean adults: The Korean Genome and Epidemiology Study (KoGES). Br. J. Nutr..

[57] Feng W., Ao H., Peng C. (2018). Gut microbiota, short-chain fatty acids, and herbal medicines. Front. Pharmacol..

[58] Chen H., Zeng F., Li S., Liu Y., Gong S., Lv X., Zhang J., Liu B. (2019). Spirulina active substance mediated gut microbes improve lipid metabolism in high-fat diet fed rats. J. Funct. Foods.

[59] Guarner F. (2014). Decade in review-gut microbiota: The gut microbiota era marches on. Nat. Rev. Gastroenterol. Hepatol..

[60] McLoughlin R.F., Berthon B.S., Jensen M.E., Baines K.J., Wood L.G. (2017). Short-chain fatty acids, prebiotics, synbiotics, and systemic inflammation: A systematic review and meta-analysis. Am. J. Clin. Nutr..

[61] Li T., Teng H., An F., Huang Q., Chen L., Song H. (2019). The beneficial effects of purple yam (*Dioscorea alata* L.) resistant starch on hyperlipidemia in high-fat-fed hamsters. Food Funct..

[62] Hu L., Tian K., Zhang T., Fan C., Zhou P., Zeng D., Zhao S., Li L., Smith H.S., Li J. (2019). Cyanate induces oxidative stress injury and abnormal lipid metabolism in liver through Nrf2/HO-1. Molecules.

[63] Li M., Wei Y., Cai M., Gu R., Pan X., Du J. (2021). Perilla peptides delay the progression of kidney disease by improving kidney apoptotic injury and oxidative stress and maintaining intestinal barrier function. Food Biosci..

[64] Namaei P., Ghadiri F., Jamali R., Azimi A.R., Shabestari H.R.F., Vahabizad F. (2022). Evaluation of liver injury in multiple sclerosis patients receiving pulsed steroid therapy. Mult. Scler. Relat. Dis..

[65] Bhatt D.P., Houdek H.M., Watt J.A., Rosenberger T.A. (2013). Acetate supplementation increases brain phosphocreatine and reduces AMP levels with no effect on mitochondrial biogenesis. Neurochem. Int..

[66] Jiang L., Krystal J.H., Mason G.F., Jiang L., Gulanski B.I., Feyter H.M.D., Weinzimer S.A., Pittman B., Guidone E., Koretski J. (2013). Increased brain uptake and oxidation of acetate in heavy drinkers find the latest version: Increased brain uptake and oxidation of acetate in heavy drinkers. J. Clin. Investig..

[67] Frost G., Sleeth M.L., Sahuri-arisoylu M., Lizarbe B., Cerdan S., Brody L., Anastasovska J., Ghourab S., Hankir M., Zhang S. (2014). The short-chain fatty acid acetate reduces appetite via a central homeostatic mechanism. Nat. Commun..

[68] Psichas A., Sleeth M.L., Murphy K.G., Brooks L., Bewick G.A., Hanyaloglu A.C., Ghatei M.A., Bloom S.R., Frost G. (2014). The short chain fatty acid propionate stimulates GLP-1 and PYY secretion via free fatty acid receptor 2 in rodents. Int. J. Obesity.

[69] Tolhurst G., Heffron H., Lam Y.S., Parker H.E., Habib A.M., Diakogiannaki E., Cameron J., Grosse J., Reimann F., Gribble F.M. (2012). Short-chain fatty acids stimulate glucagon-like peptide-1 secretion via the g-protein-coupled receptor FFAR2. Diabetes.

[70] Lu Y., Fan C., Li P., Lu Y., Chang X., Qi K. (2016). Short Chain fatty acids prevent high-fat-diet-induced obesity in mice by regulating G protein-coupled receptors and gut microbiota. Sci. Rep..

[71] Den Besten G., Bleeker A., Gerding A., Van Eunen K., Havinga R., Van Dijk T.H., Oosterveer M.H., Jonker J.W., Groen A.K., Reijngoud D.-J. (2015). Short-chain fatty acids protect against high-fat diet-induced obesity via a PPARγ-dependent switch from lipogenesis to fat oxidation. Diabetes.

[72] Sahuri-Arisoylu M., Brody L.P., Parkinson J.R., Parkes H., Navaratnam N., Miller A.D., Thomas E.L., Frost G., Bell J.D. (2016). Reprogramming of hepatic fat accumulation and ‘browning’ of adipose tissue by the short-chain fatty acid acetate. Int. J. Obesity.

[73] Kimura I., Inoue D., Maeda T., Hara T., Ichimura A., Miyauchi S. (2011). Short-chain fatty acids and ketones directly regulate sympathetic nervous system via G protein-coupled receptor 41. Proc. Natl. Acad. Sci. USA.

[74] Bellahcene M., O’Dowd J.F., Wargent E.T., Zaibi M.S., Hislop D.C., Ngala R.A., Smith D.M., Cawthorne M.A., Stocker C.J., Arch J.R. (2013). Male mice that lack the G-protein-coupled receptor GPR41 have low energy expenditure and increased body fat content. Br. J. Nutr..

[75] Andrade-Oliveira V., Amano M.T., Correa-Costa M., Castoldi A., Felizardo R.J.F., Almeida D.C.d., Bassi E.J., Moraes-Vieira P.M., Hiyane M.I., Rodas A.C.D. (2015). Gut bacteria products prevent AKI induced by ischemia-reperfusion. J. Am. Soc. Nephrol..

[76] Vadder F.D., Kovatcheva-Datchary P., Goncalves D., Vinera J., Mithieux G. (2014). Microbiota-generated metabolites promote meta-bolic benefits via gut-brain neural circuits. Cell.

[77] Vily-Petit J., Soty M., Silva M., Micoud M., Bron C., Guérin-Deremaux L., Mithieux G. (2023). Improvement of energy metabolism associated with NUTRIOSE^®^ soluble fiber, a dietary ingredient exhibiting prebiotic properties, requires intestinal gluconeogenesis. Food Res. Int..

[78] Saad M.J.A., Santos A., Prada P.O. (2016). Linking gut microbiota and inflammation to obesity and insulin resistance. Physiology.

[79] Gao Z., Yin J., Zhang J., Ward R.E., Martin R.J., Lefevre M., Cefalu W.T., Ye J. (2009). Butyrate improves insulin sensitivity and increases energy expenditure in mice. Diabetes.

[80] Christiansen C.B., Gabe M.B.N., Svendsen B., Dragsted L.O., Rosenkilde M.M., Holst J.J. (2018). The impact of short-chain fatty acids on GLP-1 and PYY secretion from the isolated perfused rat colon. Am. J. Physiol.-Gastrointest. Liver Physiol..

[81] Fan Y., Pedersen O. (2021). Gut microbiota in human metabolic health and disease. Nat. Rev. Microbiol..

[82] Li H., Zhou D., Gan R., Huang S., Zhao C., Shang A., Xu X., Li H. (2021). Effects and mechanisms of probiotics, prebiotics, synbiotics, and postbiotics on metabolic diseases targeting gut microbiota: A narrative review. Nutrients.

[83] Cai M., Chen S., Ma Q.H., Yang K., Sun P.L. (2019). Isolation of crude oligosaccharides from *Hericium erinaceus* by integrated membrane technology and its proliferative activity. Food Hydrocoll..

[84] Das B., Sahoo R.N., Pargal S., Krishna G., Verma R., Chinnusamy V., Sehgal V.K., Gupta V.K., Dash S.K., Swain P. (2018). Quantitative monitoring of sucrose, reducing sugar and total sugar dynamics for phenotyping of water-deficit stress tolerance in rice through spectroscopy and chemometrics. Spectrochim. Acta A.

