# Supplementary material for: Alleviation of High-Fat Diet-Induced Hyperlipidemia in Mice by Stachys sieboldii Miq. Huangjiu via the Modulation of Gut Microbiota Composition and Metabolic Function
Source: Foods. 2024 Jul 26;13(15):2360. doi: 10.3390/foods13152360 (PMC11312184; doi:10.3390/foods13152360)
Supplement: Supplementary file 1 [file foods-13-02360-s001.zip › foods-3099601-supplementary.pdf]

Supplementary Material

Alleviation of High-Fat Diet-Induced Hyperlipidemia in Mice by *Stachys sieboldii* Miq. Huangjiu via the Modulation of Gut Microbiota Composition and Metabolic Function

Jingzhang Geng [1,2], Yunxia Wu [1], Honglei Tian [1] and Jianwei Dong [1,*]

[1] Shaanxi Province Key Laboratory of Bio-Resources, QinLing-Bashan Mountains Bioresources Comprehensive Development C. I. C., Qinba State Key Laboratory of Biological Resources and Ecological Environment, Shaanxi University of Technology, 1 East 1st Ring Road, Hanzhong 723001, China; gengjingzhang@163.com (J.G.); zshanshan2024@163.com (Y.W.); thl0993@sina.com (H.T.)

[2] School of Life Science and Technology, Xi'an Jiaotong University, Xianning West Road, Xi'an 710049, China

* Correspondence: jwdong@snut.edu.cn

Table of Contents

Subject	Page
**Determination of the main ingredients in CSCHJ**	**S-3**
**Table S1.** Comparison of daily food intake of mice in each group before and after intervention	**S-3**
**Table S2.** FBG concentration of mice in each group before and after intervention	**S-4**
**Table S3**. Sample sequencing quantity statistics	**S-4**
**Figure S1** Alpha diversity dilution curve	**S-5**
**References**	**S-5**

Determination of the main ingredients in CSCHJ

The alcohol concentration of Huangjiu was determined by potassium dichromate-DNS colorimetric method. The total acid in Huangjiu was determined by acid-base titration, and the final result was calculated as lactic acid. The amino nitrogen in Huangjiu was determined by potentiometric titration. The total sugar and reducing sugar content in Huangjiu samples were determined using the phenol-sulfuric acid method [1] and the 3,5-dinitrosalicylic acid colorimetry method [2], respectively, and the polysaccharide content could be calculated as follows:

$$\text{polysaccharide content} = \text{total sugar content} - \text{reducing sugar content} \quad (S1)$$

Table S1 Daily food intake of mice in each group before and after intervention

Groups	Daily food intake(g)				
	Before intervention	After intervention			
		1 week	2 weeks	3 weeks	4 weeks
NC	5.77±0.19	5.62±1.03	5.65±0.68	5.01±0.73	5.44±0.71
MG	9.96±0.33$^{\#\#}$	9.86±0.54$^{\#\#}$	11.09±0.61$^{\#\#}$	10.43±1.05$^{\#\#}$	10.08±0.63$^{\#\#}$
AG	9.93±0.61	8.07±1.55	8.88±0.43**	9.12±0.43	9.70±0.78
SH-L	9.43±0.26	7.5±1.16**	8.03±1.29**	7.52±0.29**	7.46±1.04**
SH-M	9.36±0.25	7.07±0.89**	6.96±0.99**	6.36±0.75**	6.07±0.87**
SH-H	9.51±0.59	6.96±1.62**	7.06±1.23**	5.99±0.63**	5.34±1.01**

Note: $^{\#}P$<0.05 and $^{\#\#}P$<0.01 versus the normal control group (NC). $^{*}P$<0.05 and $^{**}P$<0.01 versus the model group (MG).

Table S2 FBG concentration of mice in each group before and after intervention

Groups	FBG (mmol/L)	
	Before intervention	After intervention

		1 week	2 weeks	3 weeks	4 weeks
NC	4.98±0.64	5.47±0.43	5.36±0.57	5.11±0.81	5.47±0.59
MG	7.61±0.76$^{\#\#}$	8.03±0.80$^{\#\#}$	8.14±0.77$^{\#\#}$	8.03±0.62$^{\#\#}$	8.26±0.33$^{\#\#}$
AG	7.44±0.86	8.11±0.91	8.23±1.14	8.39±0.86	8.70±0.42
SH-L	7.24±0.46	7.87±0.69	7.80±0.89	7.19±1.03	7.01±0.51**
SH-M	7.60±0.94	8.07±0.53	7.58±0.58	6.75±0.29**	6.28±0.33**
SH-H	7.18±0.80	7.92±0.48	7.51±0.61	6.63±0.64**	6.25±0.30**

Note: $^{\#}P<0.05$ and $^{\#\#}P<0.01$ versus the normal control group (NC). $^{*}P<0.05$ and $^{**}P<0.01$ versus the model group (MG).

Table S3 Sample Sequencing Quantity Statistics

Groups	Raw tags	Valid Tags	Valid%	Q20%	Q30%
NC1	82044	73459	89.54	97.37	92.89
NC2	87069	74889	86.01	97.43	92.99
NC3	80077	69015	86.19	97.46	93.07
MG1	81759	71951	88.00	97.48	93.07
MG2	83300	73805	88.60	96.75	91.12
MG3	84151	74839	88.93	97.38	92.85
AG1	84222	74182	88.08	97.29	92.52
AG2	86524	70883	81.92	96.79	91.32
AG3	84088	74952	89.14	97.14	92.36
SH-L1	86643	76905	88.76	97.01	91.996
SH-L2	86890	75530	86.93	97.40	93.02
SH-L3	84003	73718	87.76	97.44	92.98
SH-M1	81691	76365	93.48	97.31	92.72
SH-M2	81556	72073	88.37	97.46	92.87
SH-M3	86472	73460	84.95	96.84	91.43
SH-H1	86157	69341	80.48	97.22	92.69
SH-H2	80921	67221	83.07	97.41	93.06
SH-H3	84504	70313	83.21	97.24	92.59

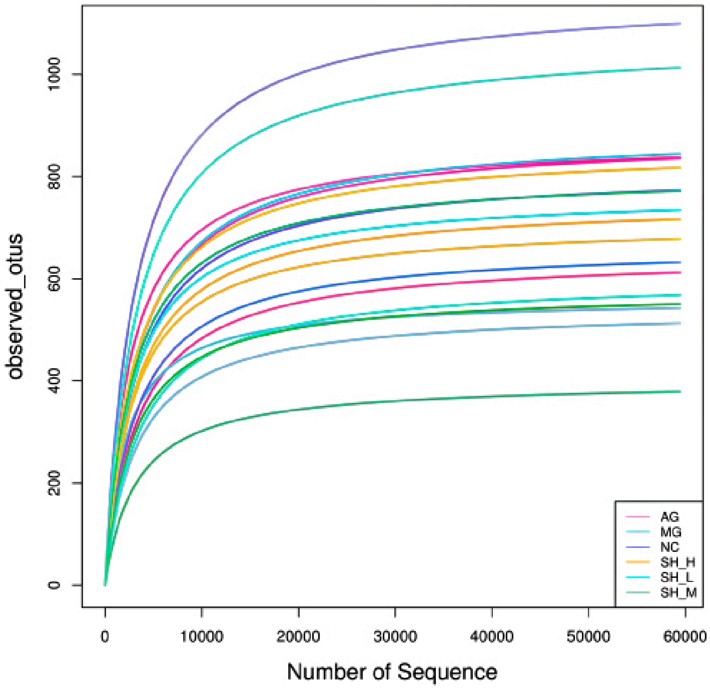

Figure S1 Alpha diversity dilution curve

References

[1] Cai, M.; Chen, S.; Ma, Q.H.; Yang, K.; Sun, P.L. Isolation of crude oligosaccharides from Hericium erinaceus by integrated membrane technology and its proliferative activity. *Food Hydrocolloids*, **2019**, *95*, 426–431.

[2] Das, B.; Sahoo, R.N.; Pargal, S.; Krishna, G.; Verma, R.; Chinnusamy, V.; Sehgal, V.K.; Gupta, V.K.; Dash, S.K.; Swain, P. Quantitative monitoring of sucrose, reducing sugar and total sugar dynamics for phenotyping of water-deficit stress tolerance in rice through spectroscopy and chemometrics. *Spectrochim. Acta A*, **2018**, *192*, 41–51.
